# Different next-generation sequencing pipelines based detection of tumor DNA in cerebrospinal fluid of lung adenocarcinoma cancer patients with leptomeningeal metastases

**DOI:** 10.1186/s12885-019-5348-3

**Published:** 2019-02-12

**Authors:** Mengxi Ge, Qiong Zhan, Zhenzhen Zhang, Xiaoyu Ji, Xinli Zhou, Ruofan Huang, Xiaohua Liang

**Affiliations:** 1 Department of Oncology, Huashan Hospital Fudan University, Shanghai, China; 2 Singlera Genomics Inc., Shanghai, China

**Keywords:** Lung adenocarcinoma cancer, Leptomeningeal metastases, Cerebrospinal fluid, CSF cells, NGS pipelines

## Abstract

**Background:**

The nucleic acid mutation status in intracranial metastasis is markedly significant clinically. The goal of the current study was to explore whether the tumor-associated mutations can be detected by different next-generation sequencing (NGS) pipelines in paired cerebrospinal fluid (CSF) and plasma samples from lung adenocarcinoma (LAC) patients with leptomeningeal metastases (LM).

**Methods:**

Paired CSF cell free DNA (cfDNA), CSF cells, plasma and formalin-fixed and paraffin-embedded (FFPE) samples of primary tumors were collected from 29 LAC patients with LM to detect the mutations by different NGS pipelines.

**Results:**

DNA libraries were generated successfully for 79 various samples in total for NGS sequencing, of which mutations were detected in 7 plasma samples (24.14%), 12 CSF cfDNA samples (66.67%), and 10 CSF cells (76.9%) samples. For the 26 patients with detected mutations, 8/26(30.77%) had mutations in plasma, which was significantly lower than that those from CSF cfDNA (12/15, 80.00%), CSF cells (10/11, 90.91%) and FFPE samples (13/17, 76.47%). When the input DNA of CSF cells was less than 20 ng, the cHOPE pipeline of NGS identified the most mutations for epidermal growth factor receptor (EGFR).

**Conclusions:**

NGS-based detection of mutations in cfDNA or cells from CSF provided more information than from plasma samples from LAC patients with LM. In addition, the cHOPE pipeline performed better than the other three NGS pipelines when input DNA from CSF cells was low.

**Electronic supplementary material:**

The online version of this article (10.1186/s12885-019-5348-3) contains supplementary material, which is available to authorized users.

## Background

Lung cancer is one of the most common malignant tumor in the world. Leptomeningeal metastasis which predicts poor prognosis occurs in about 5% lung cancer patients [1]. Genomic characterization of tumor is crucial for treatments. Specifically, more than half lung cancer patients were detected to have acquired EGFR T790 M mutation after they were treated with Gefitinib or Erlotinib [2]. It has become a consensus that lung cancer patients with tumor progression should carry out a re-biopsy to adjust the treatment [3]. However, most lung cancer patients with leptomeningeal metastases cannot get leptomeningeal tumor tissue for DNA mutation testing [4]. Liquid biopsy based on the detection of circulating tumor DNA in plasma also played a limited role for patients with intracranial lesions [5, 6].

It was reported that tumor-associated DNA could be detected in the CSF samples of cancer patients with central nervous system (CNS) tumor using NGS sequencing [6, 7]. Previous studies reported that DNA in brain tumors showed more common mutations with those in CSF when compared to plasma DNA in cancer patients [5], and CSF cfDNA could reveal the unique genetic profiles of leptomeningeal metastases in EGFR-mutated non-small cell lung cancer (NSCLC) [8]. These findings suggest that CSF is a potential source of tumor-derived DNA in lung cancer patients with leptomeningeal metastases. Jiang et al. [9] showed that tumor mutations in leptomeningeal metastases of NSCLC could be detected from CSF tumor cells, which demonstrated that we could get the tumor-related genetic information from both supernatant and cell pellets of the CSF samples of lung cancer patients with leptomeningeal metastases.

In this study, different detection pipelines were utilized for CSF cfDNA, cells, plasma and FFPE samples of primary lung cancer to identify sequence mutations. It aimed to find out whether CSF as liquid biopsy samples in a clinical setting can be substantiated and can be considered as an excellent substitute to primary cancer tissue.

## Methods

### Patients and sampling

Twenty-nine pathologically confirmed lung cancer patients with leptomeningeal metastases were enrolled in this cohort (Table 1) between Jan 1, 2016 and June 30, 2016 in the Department of Oncology, Huashan Hospital Fudan University. The inclusion criteria were positive results of malignant cells detection in CSF. We collected baseline variables such as age at diagnosis, sex and recorded type of EGFR mutation in primary lesions by amplification refractory mutation system. The present study was approved by the Ethics Committee of Huashan Hospital Fudan University (No. KY2017–010). Informed consent was obtained from all individual participants included in the study.

**Table 1:** Demographic data of 29 patients and next-generation sequencing results of different samples

patient ID	Age(years)	Sex	Mutations detected by NGS in different Sample types (mutation allele fractions)				EGFR status in primary tumor by ARMS
			plasma ctDNA	CSF cfDNA	CSF cell	FFPE of primary cancer	
#1	59	M	EGFR L747_E749del (42.5%)	EGFR L747_E749del (98.1%)	EGFR L747_E749del (37.8%)	EGFR L747_E749del (29.2%)	EGFR wild-type
#2	54	F	No mutation	ALK G689R (1.0%)	No mutation	EGFR L858R (18.8%)	EGFR L858R
#3	70	M	No mutation	No mutation	No mutation	No mutation	EGFR wild-type
#4	53	M	No mutation	EGFR E746_A750del (44.2%); TP53 R158L (97.8%)	EGFR E746_A750del (43.6%); TP53 R158L (77.0%)	EGFR E746_A750del (67.4%); TP53 R158L (40.0%)	Not available
#5	73	F	No mutation	EGFR L858R (34.4%)	ALK G689R (1.2%); EGFR L858R (7.3%)	EGFR L858R (24.1%)	Not available
#6	54	M	FGFR1 R455H (46.8%)	EGFR E746Valfs (7.3%), P753Rfs (11.4%); FGFR1 R455H (33.4%)	EGFR E746Valfs (5.9%), P753Rfs (6.1%);	Not available	EGFR wild-type
#7	48	F	EGFR G719S (0.3%)	EGFR L858R (5.3%)	EGFR L858R (2.4%)	Not available	EGFR L858R
#8	60	F	No mutation	No mutation	No mutation	Not available	Not available
#9	35	M	EGFR G719S (0.1%)	KRAS Q61L (0.1%); TP53 W53* (85.5%)	TP53 W53* (45.1%)	Not available -	Not available
#10	72	F	No mutation	EGFR L858R (51.8%)	EGFR L858R (3.8%)	Not available	EGFR L858R
#11	50	F	No mutation	EGFR L858R (41.1%); TP53 D228Valfs (87.9%)	EGFR L858R (10.7%); TP53 D228Valfs (12.8%)	Not available	EGFR L858R
#12	54	F	No mutation	TP53 V272 M (77.3%); EGFR E746_A750del (25.1%)	TP53 V272 M (26.1%); EGFR E746_A750del (19.0%)	Not available	EGFR 19del
#13	55	M	No mutation	EGFR E746_A750del (19.2%)	Not available	EGFR E746_A750del (43.2%)	EGFR 19del
#14	46	M	No mutation	No mutation	Not available	EGFR E746_A750del (88.0%)	EGFR 19del
#15	60	M	EGFR T263P (0.19%)	EGFR L858R (52.7%)	Not available	Not available	EGFR L858R
#16	67	F	No mutation	No mutation	Not available	No mutation	EGFR wide-type
#17	55	F	No mutation	No mutation	Not available	EGFR E746_A750del (48.0%); TP53 T125 M (60%)	EGFR 19del
#18	69	M	No mutation	No mutation	Not available	Not available	EGFR wide-type
#19	43	F	No mutation	Not available	EGFR E746_A750del (1.2%)	Not available	EGFR 19del
#20	48	F	No mutation	Not available	Not available	No mutation	EGFR wide-type
#21	72	F	EGFR E746_A750del (17.8%)	Not available	Not available	EGFR E746_A750del (2.7%)	EGFR 19del
#22	49	F	No mutation	Not available	Not available	EGFR E746_A750del (52.4%)	EGFR 19del
#23	49	F	TP53 N268 fs* (22.7%)	Not available	Not available	EGFR E746_A750del (34.3%); TP53 N268 fs* (20.8%)	Not available
#24	67	F	No mutation	Not available	Not available	No mutation	EGFR wide-type
#25	68	F	EGFR E746_A750del (0.6%)	Not available	Not available	EGFR E746_A750del (88.4%)	EGFR 19del
#26	43	F	No mutation	Not available	Not available	No mutation	EGFR wide-type
#27	66	M	No mutation	Not available	Not available	TP53 R280S (38.7%)	EGFR L858R
#28	72	M	No mutation	Not available	Not available	EGFR L858R (27.6%)	EGFR L858R
#29	71	F	No mutation	Not available	Not available	No mutation	EGFR 19del
Total Tests			29	18	13	19	24

About 10 ml of CSF, 10 ml blood and FFPE primary tumor tissues were collected from the enrolled patients. Two ml of CSF was used for cytological examination, and the remainder was centrifuged at 1600 g for 15 min at 4 °C to separate the supernatant and cell pellets. Blood samples were immediately centrifuged at 1600 g for 15 min at 4 °C after being sampled from patients. The supernatant was collected into a new tube for another centrifuge at 16,000 g for 10 min at 4 °C, and the second supernatant was collected as plasma samples. The supernatant of CSF (CSF cfDNA), the cell pellets of CSF (CSF cells), and the plasma were stored at − 80 °C until DNA extractions.

### DNA extraction from different samples

cfDNA from CSF supernatant and plasma were extracted using the QIAamp Circulating Nucleic Acid Kit (Qiagen, Hilden, Germany). DNA from the cell pellets of CSF were extracted using the QIAamp DNA Mini Kit (Qiagen, Hilden Germany). DNA from the FFPE tissues were extracted using the QIAamp DNA FFPE Tissue Kit (Qiagen, Hilden, Germany). All the DNA extraction experiments were handled strictly in accordance with the corresponding manufacturer’s instructions. DNA was quantified using the Qubit Fluorometer (Thermo scientific, MA, USA).

For NGS sequencing, DNA input should not be less than 30 ng and the majority of fragments were located between 150 and 180 bp for DNA from the CSF supernatant and the plasma; while DNA input should not be less than 20 ng and the majority of fragments were located above 500 bp for DNA from CSF cell pellets and the FFPE tissues.

### NGS sequencing

Different pipelines of NGS library preparation and sequencing were supplied by Singlera Genomics, Inc. Shanghai, China, for different types of samples. ddCAP pipeline was used for libraries prepared from plasma samples and CSF supernatant samples. OncoAim, cHOPE, or ddCAP-on-tissue pipelines were used for CSF cell pellets samples. OncoAim pipeline was used for FFPE samples. DNA library preparation and NGS sequencing were performed according to manufacturer’s recommended protocols. Generally, target regions were enriched by either targeted captureor multiple PCR 150 bp paired-end sequencing was performed on Illumina platforms including NextSeq 500, Hiseq 4000, MiSeq, or MiniSeq (Illumina Inc., San Diego, CA, USA). Features of these NGS pipelines were showed in Table 2.

**Table 2:** Feathers of the next-generation sequencing pipelines used for different sample types

	ddCAP	ddCAP on tissue	cHOPE	OncoAim
Number of Genes	10	10	59	59
Targets	all exons	all exons	hotspots	hotspots
Enrich method	targeted capture	targeted capture	multiple PCR	multiple PCR
DNA input	5~30 ng	50 ng	5~20 ng	10~20 ng
Instruments	NextSeq	NextSeq	NextSeq/miniSeq	NextSeq/miniSeq/MiSeq

### ddPCR test for conflicting results in one sample

The CSF cells sample in patient #12 was analyzed by drops digital PCR (ddPCR) for the mutation of EGFR E746_A750del, which showed conflicting statuses between the results of cHOPE and ddCAP-on-tissue pipelines. The ddPCR experiment was conducted using the QX200TM ddPCR system (Bio-Rad, CA, USA), and ddPCR Supermix and primer/probes for EGFR from BioRad were used.

### Data analysis and statistics

Bioinformatical analyses were performed for results from each pipeline. Briefly, sequencing reads were quality-filtered, assembled and aligned against the reference genome hg19/GRCh37. Unique reads derived from GATK were used for variant calling. The minimum confidence threshold for variant and insertion/deletion (indel) calling was set to 0.05 (5%). Variation annotation, effect prediction and clinical practice guidance were integrated into the pipelines through vcf files.

Comparisons among the detection rates of plasma, CSF cfDNA, CSF cells, and FFPE samples were performed by Fisher’s exact test using SPSS v19.0 software. Significance was assumed for a *p*-value of less than 0.05. Column graphs were generated using Graphpad Prism (version 5.0a) software.

## Results

### NGS libraries of different types of samples

Twenty-nine patients with an average age of 55 (range from 35 to 73) were enrolled in our study, of which 18 were female and 11 were male. All the enrolled patients had LAC. Patients’ information was shown in Table 1. CSF supernatant of a volume of 1.2 to 12 ml was collected from all 29 patients, of which 18 NGS libraries were generated successfully from those between 2.5 to 12 ml. Cell pellets from CSF were collected from 27 patients, of which 13 NGS libraries were generated successfully from samples whose volume ranged from 0.2 to 2.6 ml. Plasma samples (1~10.5 ml) and FFPE samples (1~39 Slices) were collected from 29 and 19 patients respectively, and NGS libraries were generated successfully for all these samples (Table 3). In total DNA libraries were prepared successfully for a total of 79 samples of various types for sequencing.

**Table 3:** Different samples enrolled in the study

Sources	Number	Start ml	DNA yield (ng)	SampleNum	Library QC
CSF supernatant	29	1.2~8.6	0.00~7.70	111	failed
		2.5~12	9.74~642	18	pass
CFS cell pellet	29	1.2~8.6	3.28~15.6	16	failed
		2.5~8.6	5~48.5	13	pass
Plasma	29	1~10.5	13~162	29	pass
FFPE	19	\	27.42~439.6	19	pass

### NGS results by different pipelines (Table 1 and Table 4)

A total of 29 plasma samples were collected, and the input DNA for library preparation ranged from 13 ng to 150 ng. Mutations were detected in only 7/29 (24.14%) plasma samples. NGS library of CSF cfDNA were generated for 18 patients with input DNA ranging from 9.5 ng to 50.5 ng. Mutations were detected in 12 of 18 (66.67%) CSF cfDNA samples.

**Table 4:** Mutations in CSF cell samples detected with different pipelines

Patients	CFS cell Mutation sites/ (DNA input)			
	cHOPE	OncoAim	ddCAP	ddCAP on Tissue
#1	EGFR L747_E749del	NA	NA	NA
	(8 ng)			
#2	No mutation	NA	No mutation	NA
	(11 ng)		(20 ng)	
#3	No mutation	NA	NA	NA
	(14 ng)			
#4	EGFR E746_A750del; TP53 R158L	NA	TP53 R158L	NA
	(9 ng)		(30 ng)	
#5	ALK G689R; EGFR L858R (20 ng)	NA	NA	EGFR L858R (50 ng)
#6	EGFR E746Valfs, P753Rfs (20 ng)	EGFR E746_P753 > VS (20 ng)	NA	NA
#7	NA	NA	NA	EGFR L858R
				(50 ng)
#8	NA	NA	No mutation	NA
			(7 ng)	
#9	TP53 W53	NA	NA	TP53 W53
	(5 ng)			(50 ng)
#10	NA	NA	EGFR L858R	NA
			(30 ng)	
#11	EGFR L858R; TP53 D228Valfs	EGFR L858R	NA	NA
	(8 ng)	(20 ng)		
#12	TP53 V272 M; EGFR E746_A750del	NA	NA	TP53 V272 M;
	(10 ng)			(50 ng)
#19	NA	NA	NA	EGFR E746_A750del
				(50 ng)

*NA* not available

We used different panels based on the quantity of DNA we extracted from the 13 CFS cell samples, and in 10/13(76.9%) samples we identified positive mutations. Samples having over 50 ng extracted DNA could be sequenced using all available pipelines, including ddCAP-on-Tissue, which was specialized for FFPEs sample in this study.

When the input DNA was less than 20 ng, the cHOPE pipeline was capable of identifying the largest amount of mutations. Indeed, seven individuals’ CSF-cell samples were analyzed using both cHOPE and a non-cHOPE pipeline. Among them 4 individuals (#5, #4, #11 and #12) had more mutations detected by cHOPE than the non-cHOPE pipelines. Two individuals (#2 and #9) had identical mutations identified by the two pipelines. The remainder (#6) was shown to have two mutations in EGFR, E746Valfs and P753Rfs, based on cHOPE pipeline, whereas a complex deletion was identified by OncoAim. In summary, mutation discoveries in CFS cells samples may yield different results due to different detection panels.

### EGFR status in the CSF cells samples for patients #12

In the CSF-cell sample from patient #12, conflicting results were obtained from 2 different NGS pipelines (Table 4). EGFR E746_A750del was identified by the cHOPE pipeline, whereas EGFR gene was shown to be wild type by the ddCAP –on-tissue pipeline. We further analyzed patient #12’s sample by ddPCR, which also identified E746_A750del mutation (8 copies/μl) in the EGFR gene (Additional file 1: Figure S1), confirming the results from cHOPE pipeline to be more reliable than those from ddCAP-on tissue.

### Tumor DNA detected in different samples

Most mutations detected in this study were located in the genes EGFR and TP53. Mutations detected in the plasma and CSF samples were also detected in the FFPE samples except the ALK G689R (CSF cfDNA of #2, and CSF cell of #5) and KRAS Q61L (CSF cfDNA of #9).

**Fig. 1:**
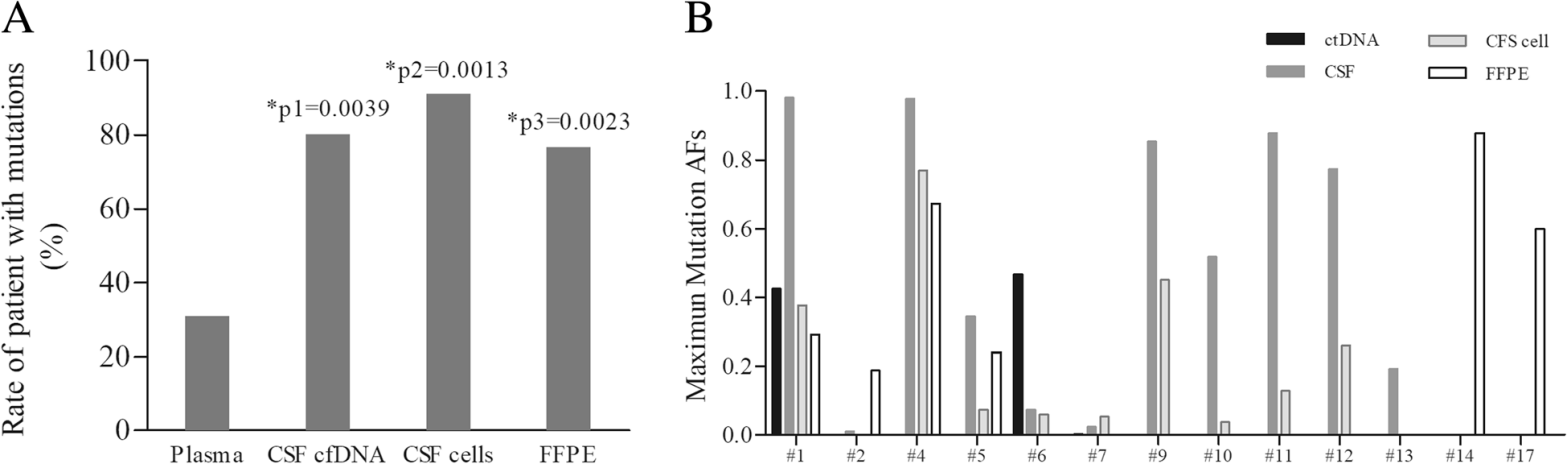
Detection rates and mutation allele fractions of different kinds of samples **a**. The difference of detection rates among 4 kinds of samples. Y- axis means No. of sample with mutations divided by No. of samples tested. p1, plasma vs. CSF cfDNA; p2, plasma vs. CSF cells; p3, plasma vs. FFPE. **b.** The maximum mutation allele fractions of the different kinds of samples in patients from which 3 or 4 kinds of sample were collected

In all 29 patients, 12 (41.38%) patients showed same results between at least two different types of samples. In the 16 patients with 3–4 types of samples, only 4 (25%) showed identical results among various samples (#1, #3, #8 and #16). No mutation was detected in the plasma, CSF or FFPE samples of patient #3, #8 and #16 (Table 1). We took these 3 individuals as negative samples to avoid statistical errors. For the other 26 patients with detected mutations, 8 (30.77%) had mutations in plasma, which was significantly lower (*P* < 0.05, Fig. 1a) than those having mutations in CSF cfDNA (12/15, 80.00%), CSF cells (10/11, 90.91%) and FFPE samples (13/17, 76.47%). The detection rates were of no significant difference between the CSF cfDNA, CSF cells and FFPE samples (*P* = 0.622).

With regard to EGFR mutations, 12 of all 13 CSF cells had the identical mutations as those in CSF cfDNA. The CSF cells from the individual (patient #12) without matching CSF cfDNA sample had a mutation of EGFR E746_A750del, which was in agreement to the EGFR 19del identified in the primary tumor by the ARMS technology (Table 1).

The average maximum allele fractions (AFs) of different sample types in an ascending order were plasma, CSF cells, FFPE tissues and CSF cfDNA, which were 13.19% (0.1%~ 46.8%, 10 mutations), 20.18% (01.19%~ 76.26%, 15 mutations), 42.72% (2.7%~ 88.4%, 16 mutations) and 49.62% (0.13%~ 98.13%, 19 mutations), respectively. (Fig. 1b).

## Discussion

In this study we used various DNA mutation detection pipelines for 4 types of biospecimens, and successfully generated NGS libraries from plasma (1~5.5 ml), CSF cfDNA (2.5~12 ml), CSF cells (0.2~2.6 ml) and FFPE sample. The input DNA for cHOPE pipeline was as low as about 5 ng. Both cHOPE and OncoAim pipelines utilized multiple PCR as enrich technology to detect tumor-associated hotspots. However, they were designed for different sample types, of which cHOPE was specialized for cfDNA while OncoAim was for DNA from FFPE sample. The other 2 NGS pipelines, ddCAP and ddCAP-on-Tissue, used target capture as enrichment technology to scan all the exons of 10 tumor-associated genes. The input DNA of ddCAP pipeline for cfDNA could be as low as 5 ng as well, whereas 50 ng DNA was required for the ddCAP-on-Tissue pipeline for DNA from FFPE sample.

In most cases that had discrepancies between CSF and plasma, CSF had more mutations detected [5, 8], which was confirmed in our study. There are several possible reasons: Firstly, because CSF circulates through the CNS and has a large interface with the CNS, it has a strong potential to carry circulating tumor DNA (ctDNA) of CNS metastases. Secondly, because there is blood-brain-barrier, DNA cannot travel between blood and CSF freely, which leads to discrepancies in DNA species in CSF and blood. Thirdly, because our cohort all had both intracranial and extracranial metastasis, spatial heterogeneity in primary lesions and CNS metastases may contribute to the discrepancies too.

Of the 19 patients who provided both plasma and CSF samples, 13 of them had mutations identified in CSF samples (cfDNA, cell pellets or both). Meanwhile, only 4 out of the 19 plasma samples had mutations identified, all of which were also discovered in the matched CSF samples. Previous research indicated that positive detection of EGFR was more sensitive in CSF than in the matched plasma [8, 10]. Our results indicate that NGS detection of CSF samples could provide more accurate and credible mutation information than plasma samples, which were consistent with the previous conclusion [10].

It was reported that more mutations could be detected in CSF cfDNA than in the paired CSF cells [8]. Due to the tumor heterogeneity and the various analysis settings in different pipelines, we did not observe same gene alternation for all the genes detected, particularly for some low AF mutations (such as ALK G689R with an AF of 1.0% and of 1.2%, and KRAS Q61L with an AF of 0.1%) and rare mutations (like FGFR1 R455H, which is not reported in COSMIC). However, similar detection rates were obtained by the sequencing for CSF cfDNA and cell samples, and the largest number of mutations were detected in those two types of sample. Especially for the EGFR gene, same mutations were identified in the cfDNA and cells of CSF sample, demonstrating the potential of using CSF cells to predict the response of EGFR-tyrosine kinase inhibitors (TKIs). This may be due to the different panels and detection methods used in the NGS library generation and sequencing. Four NGS pipelines were performed for CSF cells samples in this study. If more than 50 ng input DNA was used, all 4 panels performed well. When input DNA was less than 20 ng, cHOPE pipeline had the best performance: cHOPE pipeline identified the largest number of mutations from CSF cell samples, and most of these mutations identified by cHOPE pipeline was also discovered in the CSF cfDNA. The ddPCR results of the patients #12 confirmed the reliability of the results by cHOPE pipeline. OncoAim and ddCAP-on-tissue pipelines are designed for tissue DNA, in which fragmentation steps are contained. The fragmentation step would result in a loss of some DNA sample and miss some variations with low AF in the samples with less DNA input. For the two pipelines designed for plasma DNA, ddCAP is based on targeted capture technology for all exons of genes in the panel, whereas cHOPE is based on multiple PCR for hotspots of genes in the panel. Multiple PCR for hotspots is more focused on the variations we interested in. In general, cHOPE is the most appropriate pipeline for CSF cell samples with less than 20 ng DNA extracted.

Most mutations detected in CSF samples could be found in FFPE samples except for ALK G689R in 2 individuals and KRAS Q61L in 1 individual, demonstrating that most tumors in the brain originated from the primary lung cancer. Because these 3 CSF-only mutations had low AFs, it is unlikely that they are highly instructive in the clinical treatment in the LAC patients with LM; additionally, these mutations were likely to have emerged after the metastasis of the tumor. Although the ALK G689R nutation is labeled as a variant of undetermined significance (VUS) at present, alterations in ALK gene are involved in many malignancies including lung cancer and could mediate acquired resistance to some ALK inhibitor [11, 12]. KRAS is known to be mutated in pancreatic, colon and lung cancers [13]. Q61L was one of the hotspots in the KRAS gene and has been confirmed to influence response to EGFR antagonists for tumor patients [14]. Hence, we deduced that mutations in CSF DNA could help illustrate the gene mutation status of brain metastasis, which can further benefit the analysis of resistance mechanisms, and guide the treatment. Mutation detection of CSF cells or cfDNA improves the accuracy in accessing the feasibility of molecular targeted drug therapies, especially for EGFR-TKIs, which have been widely proven to have significantly extended the patients’ survival.

## Conclusions

NGS based detection of CSF samples of both cfDNA and cells can provide more gene information than plasma samples from LAC patients with LM. In addition, the cHOPE pipeline performed better than the other three NGS pipelines when low amount of input DNA from CSF cells was used.

## Additional file

Additional file 1: **Figure S1.** Result of EGFR E746_A750del in CSF cells of Patient #12 by ddPCR system. Y-axis means the signals of each PCR amplification, X-axis means the PCR events. Evens above the cutoff threshold represent the mutated DNA in the sample tested. (TIF 1324 kb)

## Abbreviations

AF: Allele fraction
cfDNA: Cell free DNA
CNS: Central nervous system
CSF: Cerebrospinal fluid
EGFR: Epidermal growth factor receptor
FFPE: Formalin-fixed and paraffin-embedded
LAC: Lung adenocarcinoma
LM: Leptomeningeal metastases
NGS: Next-generation sequencing
NSCLC: Non-small cell lung cancer
TKI: Tyrosine kinase inhibitors
VUS: Variant of undetermined significance
