# Supplementary figures and images for: Different next-generation sequencing pipelines based detection of tumor DNA in cerebrospinal fluid of lung adenocarcinoma cancer patients with leptomeningeal metastases
Source: BMC Cancer. 2019 Feb 12;19:143. doi: 10.1186/s12885-019-5348-3 (PMC6373107; doi:10.1186/s12885-019-5348-3)

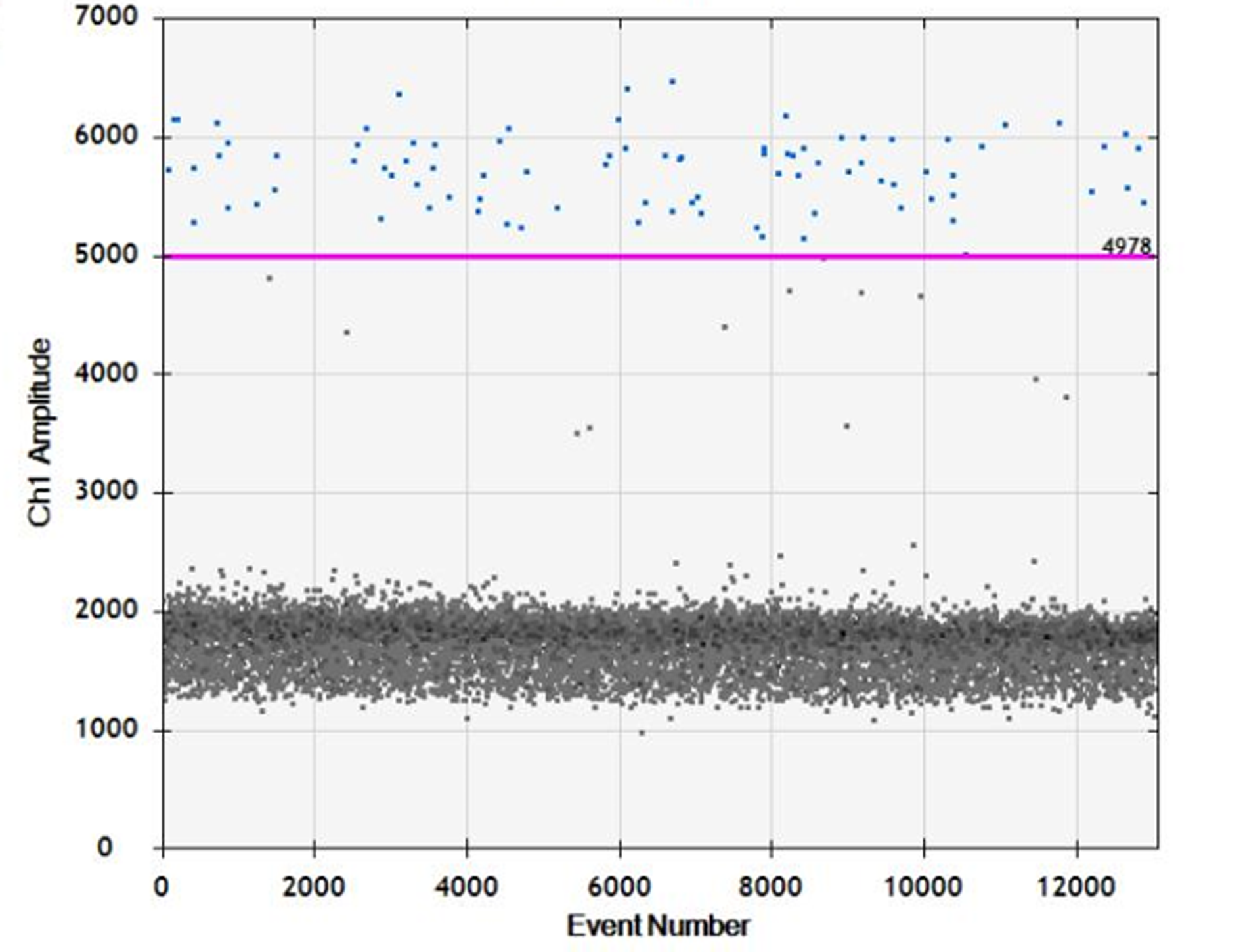

Supplement: Supplementary file 1 — Figure S1. Result of EGFR E746_A750del in CSF cells of Patient #12 by ddPCR system. Y-axis means the signals of each PCR amplification, X-axis means the PCR events. Evens above the cutoff threshold represent the mutated DNA in the sample tested. (TIF 1324 kb) [file 12885_2019_5348_MOESM1_ESM.tif]
